# Supplementary material for: Can China’s industrial policies enhance the green competitiveness of the manufacturing industry?
Source: PLoS One. 2021 Jun 30;16(6):e0253774. doi: 10.1371/journal.pone.0253774 (PMC8244902; doi:10.1371/journal.pone.0253774)
Supplement: Attachment — Submitted filename: Review comments.docx [file pone.0253774.s001.docx]

**Reviewers' comments:**

Review comments on the paper submitted to the "PLOS ONE" with the Manuscript Number: PONE-D-20-39609R1

In this study, combined with the provincial and regional panel data, the influencing degree and functional mechanism of China's industrial policies on the green competitiveness of manufacturing industry are examined empirically. Respecting functional mechanisms, supported by industrial policies, the resource allocation, innovation incentives and environmental governance have played a positive role in promoting the green competitiveness of manufacturing industry. In addition, the synergistic complementary effect between industrial policy power and market force are investigated, as well as the role of fiscal decentralization in the influencing mechanism of industrial policies.

This is a good paper from idea approaches. However, the following points need to be clarified or revised before this paper to be published;

1) *Line 70 “had”, “discusses”, Line 72 “provides”, Line76 “find” and so on. In addition,* *I can't make sense of this sentence. There are some tense and language issues making this paper hard to follow. Please carefully go through the paper again and it may have to go through a thorough proofreading by a native speaker.*
2) *I would strongly advise that the authors pay attention to linguistic alterations. The paper would benefit from some closer proofreading. It includes many linguistic errors (e.g. Line 77-78 “Tao et al. [15] Agricultural non-point source pollution control and prevention policies contributed in pollution reduction.”, Line113“a directional distance function(DDF)-based analysis model…”,Line116 “making input- or output-based…”) that at times make it difficult to follow. This problem should be addressed.*
3) *It would be better to highlight your research contribution in the introduction. This part is rarely considered in the current version, which is not attractive to me.*
4) *Unify the format of the table and retain the same decimal places.*
5) *Where are the sources of data for variables Subs, Inno and Gov in the* *search and verification of transmission channels, and the descriptive statistics for these three variables are necessary.*
6) *Whether any other control variables were included in the transmission channel analysis, and explain why you did.*
7) *Where to obtain the data of market force and FD? What about descriptive statistics? From the beginning of the data use is not clear, how to conduct more in-depth research?*
